# T-bet controls severity of hypersensitivity pneumonitis

**DOI:** 10.1186/1476-9255-8-15

**Published:** 2011-06-23

**Authors:** Hossam Aly Abdelsamed, Meena Desai, Stephanie C Nance, Elizabeth A Fitzpatrick

**Affiliations:** 1 University of Tennessee Health Science Center, Dept. of Microbiology, Immunology and Biochemistry, Memphis, TN 38163, USA

## Abstract

Hypersensitivity Pneumonitis (HP) is an interstitial lung disease that develops following repeated exposure to inhaled environmental antigens. The disease is characterized by alveolitis, granuloma formation and in some patients' fibrosis. IFNγ plays a critical role in HP; in the absence of IFNγ granuloma formation does not occur. However, recent studies using animal models of HP have suggested that HP is a Th17 disease calling into question the role of IFNγ. In this study, we report that initially IFNγ production is dependent on IL-18 and the transcription factor T-bet, however as the disease continues IFNγ production is IL-18-independent and partially T-bet dependent. Although IFNγ production is required for granuloma formation its role is distinct from that of T-bet. Mice that are deficient in T-bet and exposed to *S. rectivirgula *develop more severe disease characterized by an exacerbated Th17 cell response, decreased Th1 cell response, and increased collagen production in the lung. T-bet-mediated protection does not appear to be due to the development of a protective Th1 response; shifting the balance from a Th17 predominant response to a Th1 response by inhibition of IL-6 also results in lung pathology. The results from this study suggest that both Th1 and Th17 cells can be pathogenic in this model and that IFNγ and T-bet play divergent roles in the disease process.

## Background

Hypersensitivity Pneumonitis (HP) or extrinsic allergic alveolitis is an interstitial lung disease that develops following repeated exposure to inhaled environmental antigens [1-3]. The disease is characterized by alveolitis and granuloma formation and with continued exposure to the inciting agent some patients develop chronic irreversible fibrosis. Once a patient progresses to the chronic form of the disease the long-term prognosis is poor. Although many individuals are exposed to these environmental antigens only approximately 5-15% of the exposed population will develop disease [1,4]. The low prevalence rate suggests that environmental and/or host genetic co-factors contribute to development of disease. In addition, there is considerable variability in disease severity and response to treatment in patients that develop HP demonstrating the complexity of the disease [reviewed in (5)].

Farmers Lung Disease is one of the most common types of HP and is caused by repeated inhalation of the thermophile *Saccharopolyspora rectivirgula*, which is commonly found in moldy hay. The animal model used to study Farmers Lung disease is the well-characterized *S. rectivirgula *mouse model [6-9]. Mice intranasally inoculated with *S. rectivirgula *for 3 days/week for 3 weeks develop an alveolitis that is initially neutrophilic but becomes more lymphocytic upon subsequent exposures. By the third week of exposures granulomas develop composed of macrophages and T cells surrounded by fibroblasts. The development of granulomas is dependent on CD4^+ ^T cells; athymic nude mice do not develop disease and CD4^+ ^T cells from sensitized mice can adoptively transfer disease to naïve recipients [10-12]. The disease is dependent on IFNγ; IFNγ knockout (KO) mice exposed to *S. rectivirgula *develop alveolitis but not granulomas nor the subsequent fibrotic response [13,14]. Together, these results led to the suggestion that HP was mediated by pathogenic Th1 cells. However, some anomalies exist suggesting that HP is a much more complex disease. Previous studies in our lab demonstrated that innate immune cell IFNγ production is sufficient for granuloma formation following exposure to *S. rectivirgula *and T cell IFNγ production is not necessary [15]. Recent studies have found that IL-17 is associated with disease severity suggesting that the Th17 response is more important than a Th1 type response in the disease. Exposure of IL-17ra^-/- ^mice to *S. rectivirgula *resulted in decreased inflammation and fibrosis compared to WT mice suggesting a pivotal role for Th17 cells and IL-17 in the disease process [16]. These results call into question the role of IFNγ and Th1 cells in disease pathogenesis in this model. IFNγ is a critical mediator of the immune response, responsible for activating macrophages, stimulating pro-inflammatory cytokines, chemokines, and adhesion molecules, and differentiation of Th1 cells. IFNγ also appears to play a role in regulating the IL-17 response; studies using murine arthritis models have demonstrated that IFNγ suppresses IL-17 production and regulates the outcome of a Th1 vs Th17 response [17-19]. The absence of IFNγ during HP, results in decreased granuloma formation which would not be expected if IFNγ was acting by inhibiting the pathogenic Th17 response. These results suggest that IFNγ plays a role in the disease process other than regulation of IL-17 and the Th17 response in HP. Many of the effects of IFNγ are mediated through induction of T-bet which is the major transcription factor regulating IFNγ production. Therefore, stimulation of T-bet expression by IFNγ results in a positive feedback loop that makes it difficult to distinguish the individual contributions of T-bet and IFNγ to a disease process.

The goal of the present study was to identify the factors involved in regulation of IFNγ during HP and determine whether the effects of IFNγ were mediated by T-bet. The results show that during the innate immune response to *S. rectivirgula*, IFNγ production is dependent on IL-18 and T-bet, whereas during later phases IFNγ production was IL-18-independent and partially T-bet dependent. Although IFNγ production is required for granuloma formation its role is distinct from that of T-bet. Mice that are deficient in T-bet and exposed to *S. rectivirgula *develop more severe disease characterized by an exacerbated Th17 cell response, decreased Th1 cell response, and increased collagen production in the lung. T-bet-mediated protection does not appear to be due to the development of a protective Th1 response; shifting the balance from a Th17 predominant response to a Th1 response by inhibition of IL-6 also results in lung pathology. The results from this study suggest that both Th1 and Th17 cells can be pathogenic in this model and that IFNγ and T-bet play divergent roles in the disease process.

## Methods

### Animals and *S. rectivirgula *exposure protocol

C57BL/6, IL-18^-/- ^and T-bet^-/- ^female mice were purchased from Jackson Laboratories (Bar Harbor, ME) at 6 weeks of age. All animals were housed in sterile micro-isolator cages with sterile food and water ad libitum and were maintained by the Division of Comparative Medicine at the University of Tennessee Health Science Center according to the guidelines of the Animal Welfare Act. The institutional animal care and use committee approved all experimental procedures. The *S. rectivirgula *(strain designation A1313 - ATCC) preparation was grown at 55°C in trypticase soy broth. The bacterial preparation was washed in endotoxin free distilled water 3 times followed by sonication and lyophilization. The lyophilized preparation was reconstituted with endotoxin free saline.

### BAL and lung cell isolation

Mice were anesthetized with isoflurane and intranasally inoculated with the indicated amount of *S. rectivirgula *for 3 days/week for 3 weeks. Bronchoalveolar lavage (BAL) was performed by intranasal injection of 1 ml of PBS into the lungs with immediate vacuum aspiration. The amount of fluid recovered was routinely around 70%. Cells were recovered from BAL fluid (BALF) by centrifugation and counted using trypan blue dye exclusion. The BALF was frozen at -80 until used in ELISA assays for cytokine and chemokine measurement. Lungs were perfused with phosphate-buffered saline (PBS) to remove blood and both lobes removed. Lung tissue was digested with collagenase (20 U/ml) and deoxyribonuclease I (40 μg/ml) for 60 minutes at 37°C. Cells were freed by disruption in a Stomacher tissue processor and then isolated by centrifugation on a discontinuous percoll gradient. Mononuclear cells were isolated at the 40/80% interface following density gradient centrifugation and used in flow cytometry.

### Flow Cytometry

Flow cytometry was performed on isolated BAL and lung cells using fluorochrome-conjugated antibodies to CD11b, Gr1, CD45, CD4, CD8, βTcR chain, and NK1.1 (BD Biosciences, San Jose, CA or ebiosciences, San Diego, CA). For neutrophil cell sorting, lung cells from mice were pooled and incubated with antibodies to CD45, Ly6G, CD11b, and NK1.1. The stained cells were sorted on a BD FACSAria and neutrophils were identified as CD45^+^/Ly6G^+^/CD1b^+^/NK1.1^-^. For intracellular cytokine staining, lung cells were prepared from individual mice and enriched for lymphocytes by removing adherent cells, and then incubated overnight with *S. rectivirgula *(5 μg) in the presence of splenic adherent cells from unexposed WT mice. The next day golgi plug (BD Biosciences) was added for 5 hrs prior to staining for flow cytometry. Some lung cells were stimulated with PMA and ionomycin for 5 hrs instead of *S. rectivirgula*. Cells were stained with antibody to IFNγ, IL-17, or isotype control antibody. A minimum of 50,000 events/sample was collected on a BD Biosciences LSRII cytometer (BD Biosciences, San Jose, CA). Expression of cell surface markers and intracellular cytokines was analyzed using DIVA software.

### Histology

The left lobe of lung was removed and fixed with neutral buffered formalin and embedded in paraffin. Eight micron sections were cut and stained with Hematoxylin and Eosin (H&E) to analyze granuloma formation.

### Collagen Content

The collagen content from the right lung lobe was determined using the Sircoll collagen assay (Biocolor, UK) as per manufacturer's instructions. Briefly, one lung lobe was homogenized in 0.5 M acetic acid containing protease inhibitors and incubated for 24 hrs at 4 C. The homogenate was centrifuged and 100 μl of the supernatant was incubated with the Sirius red dye reagent. The suspension was centrifuged, excess Sirius red dye reagent was aspirated off, and the pellet was resuspended in 0.5 M NaOH. The samples and collagen standards were read at 540 nm on a spectrophotometer. The collagen content was calculated using a standard curve generated using known concentrations of collagen.

### ELISA

Cytokines present in unconcentrated BALF were measured by ELISA (ebiosciences) according to manufacturer's instructions. Cytokine standards ranging from 3.2 pg/ml to 10,000 pg/ml were prepared to determine the concentration of cytokine in the samples. For data analysis, a curve fit was applied to the standards and the sample concentrations extrapolated from the standard curve using four-parameter logistic software (SoftMax Pro, Sunnyvale, CA).

### Real-time PCR

Total RNA was extracted from the upper right lobe of lung from individual mice using TRIZOL (Invitrogen). Contaminating genomic DNA was removed by treatment with DNA-free (Ambion, Austin, TX) according to manufacturers' directions. One μg of RNA was reverse transcribed into cDNA with the Transcriptor First Strand cDNA Synthesis Kit (Roche Applied Science, Indianapolis). Real-time PCR was performed on a Lightcycler^®^480 Real-Time PCR system (Roche Applied Science). Relative quantities of mRNA were determined using the Lightcycler^®^480 Probes Master mix and the comparative threshold cycle method. Primer sequences were designed using ProbeFinder software (Roche Applied Science) and probes chosen from the Universal Probe Library. mRNA levels for each gene were normalized to hypoxanthine guanine phosphoribosyl transferase (HGRT) and the fold increase in signal over controls was determined by the ΔΔct calculation.

### Statistics

Results are expressed as mean +/- S.D. Data were analyzed using One-way ANOVA or Student's *t*-test using GraphPad Prism statistical software (GraphPad Software, San Diego, CA). Differences were considered significant at P values of less than 0.05.

## Results

### T-bet and IL-18 contribute to IFNγ production during innate immune response

**Figure 1:**
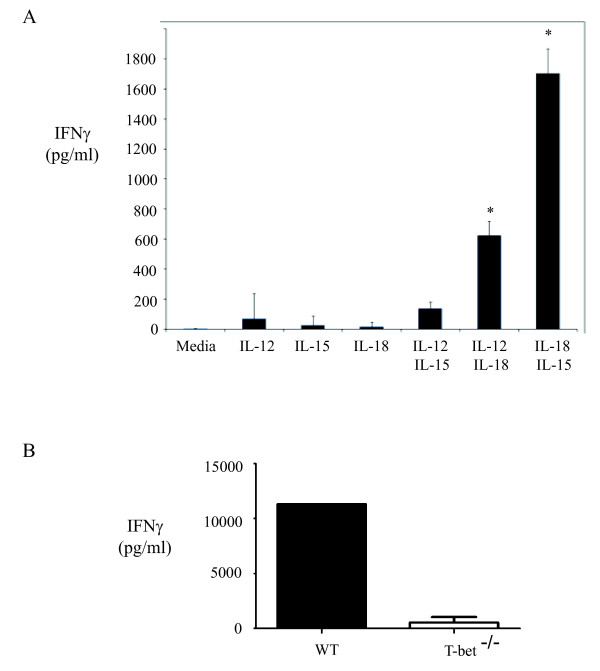
**Role of IL-18 and T-bet in IFNγ production in vitro**. A.) Neutrophils (CD45^+^/CD11b^+^/Ly6G^+^/NK1.1^-^) were sorted from the lungs of WT mice exposed to *S. rectivirgula *one time. The cells were stimulated overnight with IL-12 (10 ng/ml), IL-15 (20 ng/ml) or IL-18 (20 ng/ml) alone or in combination. Culture supernatants were collected and IFNγ measured by ELISA. B.) Spleen cells were isolated from WT or T-bet-/- mice and stimulated overnight with *S. rectivirgula *(10 μg); IFNγ was measured in cell culture supernatants by ELISA.

**Figure 2:**
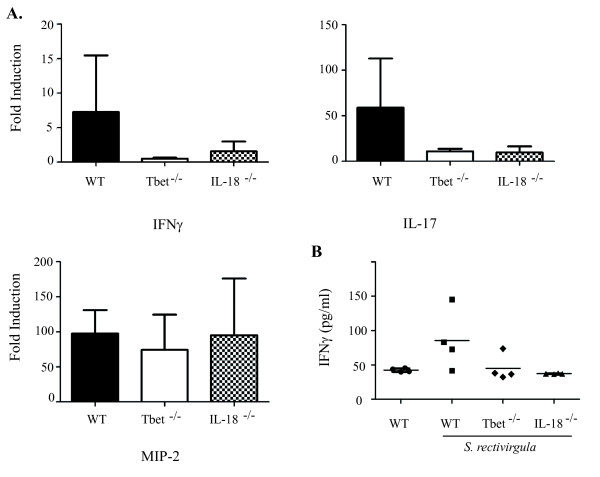
**IFNγ and IL-17 mRNA are decreased in T-bet**^**-/- **^**and IL-18R**^**-/- **^**mice following *S. rectivirgula *exposure**. C57BL/6, IL-18-/-, or T-bet-/- mice (5/group) were intranasally exposed to *S. rectivirgula *(150 μg) or saline for 3 days. A.) RNA was isolated from lungs of individual mice, reverse transcribed, and real-time PCR performed using primers specific for MIP-2, IFNγ and IL-17. The results were normalized to the housekeeping gene HPRT and expressed as fold induction over unexposed mice. B.) IFNγ was measured in the BALF of individual mice by ELISA.

Previous studies in our lab demonstrated that neutrophils produce IFNγ during the initial phase of HP and we sought to identify the cytokines responsible for stimulating neutrophil IFNγ production. Neutrophils from the lungs of mice previously exposed to *S. rectivirgula *were sorted and stimulated in vitro with IL-12, IL-15, or IL-18 alone and in combination, and IFNγ in cell culture supernatants was measured by ELISA (Figure 1A). Neutrophils did not produce IFNγ to any of the cytokines used alone, however when IL-18 was combined with either IL-12 or IL-15, significant IFNγ production was measured. The combination of IL-12 and IL-15 did not stimulate IFNγ production by neutrophils, suggesting that IL-18 was necessary for its production. IL-18 stimulates IFNγ production through the transcription factor T-bet [20,21] and we sought to determine whether *S. rectivirgula *stimulation of IFNγ production was also T-bet dependent. We stimulated spleen cells from C57Bl/6 (WT) or T-bet KO mice with *S. rectivirgula *and measured IFNγ production in the culture supernatants by ELISA (Figure 1B). The results demonstrate that *S. rectivirgula *stimulates large amounts of IFNγ from WT cells that is completely dependent on T-bet; cells from T-bet KO mice produced significantly less IFNγ compared to WT cells following stimulation. Taken together, these in vitro results suggested to us that both T-bet and IL-18 might play a role in IFNγ production during HP. To determine the role of T-bet and IL-18 in IFNγ production in vivo we exposed WT, T-bet^-/- ^and IL-18^-/- ^mice to three exposures of *S. rectivirgula *and measured cytokine production in the lungs. The results demonstrate that following exposure, WT and IL-18^-/- ^mice develop similar levels of alveolitis whereas T-bet^-/- ^mice developed a less severe alveolitis (Table 1). The overall cellular composition of the alveolitis was similar between all the groups with the exception of NK cells which were significantly reduced in the BALF from T-bet^-/- ^mice. Real time PCR was used to measure cytokine production in the lungs following exposure and the results reveal that in comparison to WT mice IFNγ mRNA is reduced in both the IL-18^-/- ^and T-bet^-/- ^groups (Figure 2A). In addition we could only detect IFNγ in the BALF of 1/5 T-bet^-/- ^mice compared to the WT group which had detectable IFNγ (Figure 2B). Surprisingly, there was also a reduction in the level of IL-17mRNA in both the T-bet^-/- ^and IL-18^-/- ^mice compared to WT mice. IL-17 stimulates MIP-2 production which in turn stimulates neutrophil recruitment. However, despite the decreased alveolitis in T-bet^-/- ^mice, the levels of MIP-2 mRNA, which is strongly induced in HP, was similar to IL-18^-/- ^and WT mice exposed to *S. rectivirgula*. These results suggest that both IL-18 and T-bet play a role in IFNγ production during the innate immune response to S. *rectivirgula*.

**Table 1:** T-bet^-/- ^mice develop less severe alveolitis compared to WT and IL-18^-/^^- ^mice following exposure to *S. Rectivirgula*

	**Alveolitis**^**a**^	**%PMN**^**b**^	**Total PMN**^**b **^**(% +/- SD)**	**Total NK**^**b **^**(+/-SD)**
**WT/unexp**	0.09 × 10^6^+/-0.04	-	-	-
**WT/*S. rectivirgula***	1.5 × 10^6^+/-0.8	73+/-5	1.1 × 10^6^+/-0.5	2.03 × 10^4^+/-0.7
**Tbet**^**-/- **^**/*S. rectivirgula***	0.5 × 10^6^+/-0.3 *	66+/-10	0.4 × 10^6^+/-0.2 *	0.05 × 10^4^+/-0.07 *
**IL-18**^**-/- **^**/*S. rectivirgula***	1.3 × 10^6^+/-0.6	78+/-3	1.0 × 10^6^+/-0.5	4.1 × 10^4^+/-2.0

C57BL/6, IL-18^-/- ^or T bet^-/- ^mice (n = 5/group) were exposed to *S. rectivirgula *(150 μg) three times and analyzed 18 hrs after last exposure.

^a ^BAL was performed and the recovered cells were counted using trypan blue dye exclusion.

^b ^Cells isolated from the BAL fluid were incubated with antibodies to various cell surface markers and analyzed by flow cytometry to determine the cellular composition of the BALF. PMNs were identified as: CD45^+^/CD11b^+^/Gr1^+^; NK cells as CD45^+^/NK1.1^+^

* *p *< 0.05 compared to WT/*S. rectivirgula *group.

### T-bet inhibits development of Th17 response

Many of the effects of IFNγ are mediated by the transcription factor T-bet. T-bet is critical for differentiation of Th1 cells, Th1 cell migration through induction of the chemokine receptor CXCR3, and has been reported to inhibit the development of a Th17 response [22-24]. To determine the role of T-bet in the disease, WT or T-bet^-/- ^mice were exposed to *S. rectivirgula *and analyzed for the development of HP at 3 and 6 weeks. T-bet^-/- ^mice developed a less severe alveolitis compared to WT mice with 6 weeks of *S. rectivirgula *exposure (Table 2). The cellular composition of the BALF in the WT and T-bet^-/- ^mice was similar with the exception of NK cells which were significantly decreased in the T-bet^-/- ^mice at 3 weeks; with 6 weeks of *S. rectivirgula *exposures the % of NK cells (and total number) in the BALF of WT mice is below 1%. The IL-18^-/- ^mice did not differ from the WT mice following *S. rectivirgula *exposure for any of the parameters analyzed (data not shown).

**Table 2:** T-bet KO mice develop less severe alveolitis following long-term exposure to *S. rectivirgula *compared to WT mice

	**Alveolitis**^**a**^		**PMN**^**b **^**(%+/-SD)**		**CD4 T cells**^**b **^**(%+/-SD)**		**CD8 T cells**^**b **^**(% +/- SD)**		**NK cells**^**b **^**(% +/- SD)**	
	WT	**Tbet **^**-/-**^	WT	**Tbet **^**-/-**^	WT	**Tbet **^**-/-**^	WT	**Tbet **^**-/-**^	WT	**Tbet**^**-/-**^
**3 wk SR**	2.2 × 10^6^+/-1.2	1.9 × 10^6^+/-1.9	72+/-6.0	70+/-14	6+/-1.5	9+/-2.5	3+/-0.7	3+/-1.1	5+/-1.3	2+/-0.1
**6 wk SR**	10.3 × 10^6^+/-2.5	3.1 × 10^6 ^+/-2.2*	88+/-3.0	91+/-1	4+/-0.4	3+/-1.0	2+/-0.3	1+/-0.4	<1	<1

C57BL/6 or T bet^-/- ^mice (n = 5/group) were exposed to *S. rectivirgula *(150 μg) three times a week for three or six weeks.

^a ^BAL was performed and the recovered cells were counted using trypan blue dye exclusion.

^b ^Cells isolated from the BAL fluid were incubated with antibodies to various cell surface markers and analyzed by flow cytometry to determine the cellular composition of the BALF. CD4^+ ^T cells were identified as βTcR^+^/CD45^+^/CD4^+^; CD8^+ ^T cells - βTcR^+^/CD45^+^/CD8^+^; PMNs - CD45^+^/CD11b^+^/Gr1^+^; NK cells - CD45^+^/NK1.1^+^

*p < 0.05 compared to WT/*S. rectivirgula *group

**Figure 3:**
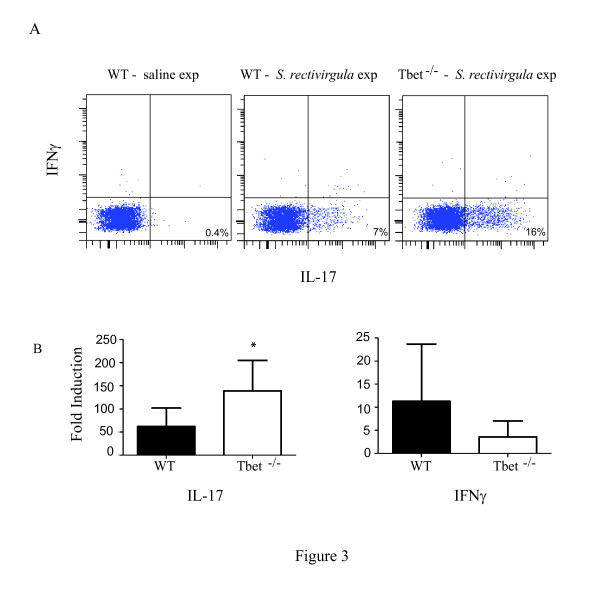
**Increase in Th17 cells in T-bet KO mice following *S. rectivirgula *exposure**. C57BL/6 or T-bet-/- mice (5/group) were intranasally exposed to *S. rectivirgula *(150 μg) or saline for 3 weeks. Lungs were removed and cells were isolated from individual mice as described in materials and methods. A) Lung cells were stimulated overnight with *S. rectivirgula *or media alone prior to intracellular cytokine staining. Lung cells from WT/saline exposed mice were pooled due to the low number of CD4+ T cells in the lungs of these mice. Cells were surface stained with antibodies to CD45, βTcR chain and CD4 followed by permeabilization and incubation with anti-IL-17 and IFNγ antibodies and run on a BD LSRII flow cytometer. The data was analyzed using DIVA software and the % of cells expressing IL-17 or IFNγ was obtained by gating on CD45^+^/βTcR^+^/CD4^+ ^T cells. B) RNA was isolated from lungs of individual mice, reverse transcribed, and real-time PCR performed using primers specific for IFNγ and IL-17 the results were normalized to the housekeeping gene HPRT and expressed as fold induction over unexposed mice. * *p *< 0.05; T-bet-/- mice exposed to *S. rectivirgula *compared to WT mice exposed to *S. rectivirgula*.

**Figure 4:**
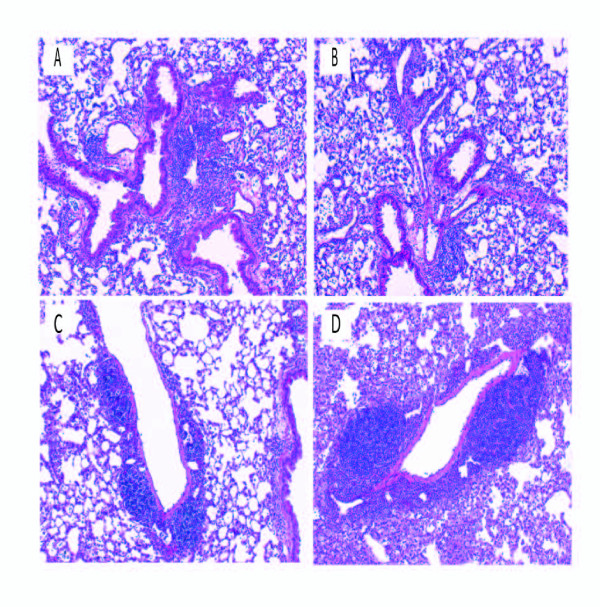
**Development of granulomas in lungs of WT and KO mice**. Representative H & E stained lung sections from WT mice (A and C), IL-6KO mice (B), and T-Bet KO mice (D), exposed to *S. rectivirgula *for 3 weeks. IL-6 KO mice exposed to *S. rectivirgula *(B) form granulomas similar to WT mice exposed to *S. rectivirgula *(A). T-bet KO mice exposed to *S. rectivirgula *(D) demonstrated more severe granuloma formation compared to WT mice exposed to *S. rectivirgula *(C). (Original magnification × 63).

**Figure 5:**
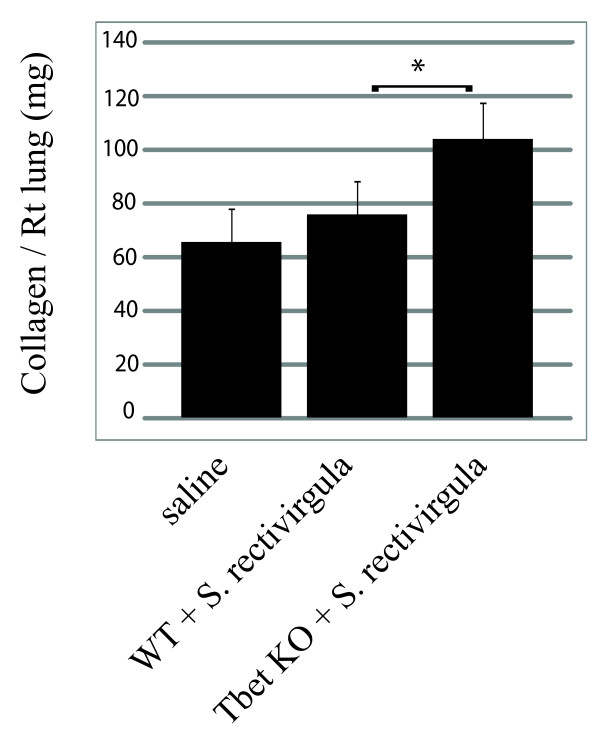
**Increased collagen in lungs of T-bet mice exposed to *S. rectivirgula ***. C57BL/6 or T-bet^-/- ^mice (5/group) were intranasally exposed to *S. rectivirgula *(150 μg) or saline for 3 weeks. The right lung lobe was removed from mice and the collagen content was determined as described in methods. The results are expressed as amount of collagen/right lung lobe and represent the average+/- SD in each group of mice. *p < 0.05; T-bet KO mice exposed to *S. rectivirgula *compared to WT mice exposed to *S. rectivirgula*.

To examine the effect of T-bet on CD4+ T cell migration into the lung flow cytometry was performed. There was no difference in the percentage of CD4+ T cells that entered the lungs of WT and T-bet^-/- ^exposed mice (WT/SR = 64%+/-5 vs T-bet^-/-^/SR = 69%+/-6). However, there was significantly less CD4+ T cells expressing the chemokine receptor CXCR3, which is induced by T-bet, in the lungs of the T-bet^-/- ^mice compared to WT mice (WT/SR = 28%+/-4 vs T-bet^-/- ^= 10+/-5; *p *< 0.05 ). To determine whether T-bet inhibits the development of Th17 cells during HP we measured the level of Th17 cells in the lungs with 3 weeks of *S. rectivirgula *exposure by intracellular cytokine staining (Figure 3A). There were almost no Th17 cells (0.4%) in the lungs of WT mice exposed to saline that responded to ex vivo *S. rectivirgula *stimulation, whereas WT mice that were exposed to *S. rectivirgula *had approximately 7% of Th17 cells in the lungs. In contrast, 16% of CD4^+ ^T cells from T-bet^-/- ^mice expressed intracellular IL-17 following stimulation with *S. rectivirgula*. We did not detect IFNγ by intracellular cytokine staining in T cells from exposed WT mice in response to ex vivo *S. rectivirgula *stimulation. However, there were approximately 3% IFNγ^+^/CD4^+ ^T cells following PMA and ionomycin stimulation suggesting there are some Th1 cells in the lungs (data not shown). Real time PCR was performed to measure the level of IL-17 or IFNγ mRNA in the lungs of WT or T-bet^-/- ^mice and the results demonstrate a significant increase in IL-17 mRNA in the T-bet^-/- ^mice compared to the WT mice exposed to *S. rectivirgula *(Figure 3B). Although there appeared to be a decrease in IFNγ mRNA expression in the T-bet^-/- ^mice compared to the WT mice this was not statistically significant. The level of IL-17 present in BALF from individual mice revealed that there was also an increase in the level of IL-17 in the BALF from T-bet^-/- ^mice exposed to *S. rectivirgula *(37+/-16 pg/ml) compared to WT mice (17+/-16 pg/ml). Similar results were observed in WT and T-bet^-/- ^mice exposed for 6 weeks (data not shown). These results demonstrate that T-bet inhibits the generation of Th17 cells during HP. To determine whether T-bet was required for granuloma formation, lungs from WT or T-bet^-/- ^mice were examined with H&E staining (Figure 4C&D). The results demonstrate that T-bet^-/- ^mice exposed to *S. rectivirgula *had granuloma formation which appeared to be increased compared to that of WT mice. The increase in severity of granuloma formation in the T-bet^-/- ^mice suggested that the disease was more severe in these mice and subsequently we would expect to see an increase in fibrosis. Therefore we measured the collagen content in the lungs of mice exposed to *S. rectivirgula *for 6 weeks (Figure 5). The results indicate that there is a significant increase in the amount of collagen from lungs of T-bet^-/- ^mice compared to the lungs of WT mice. We did not detect an increase in IL-4 or IL-13 mRNA in the T-bet^-/- ^mice compared to WT therefore the increase in severity appears to be due to the increased Th17 response and not a shift to a Th2 response (data not shown). These results suggest that T-bet plays a protective role in the disease by inhibiting the Th17 response, granuloma formation, and fibrosis.

### Th17 cells are not absolutely required for granuloma formation in HP

**Figure 6:**
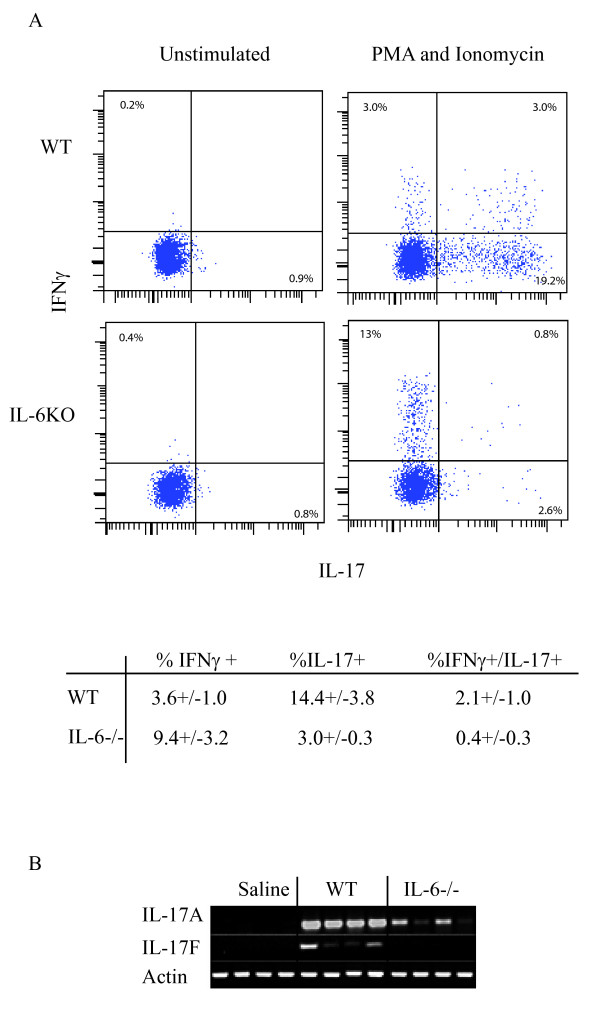
**IL-6 is required for development of Th17 cells during HP**. C57BL/6 or IL-6^-/- ^mice (5/group) were intranasally exposed to *S. rectivirgula *(150 μg) or saline for 3 weeks. Lungs were removed and cells were isolated from individual mice as described in materials and methods. A). Lung cells were stimulated with media alone or PMA and ionomycin for 4 hrs prior to intracellular cytokine staining. Cells were surface stained with antibodies to CD45, βTcR chain and CD4 followed by permeabilization and incubation with anti-IL-17 and IFNγ antibodies and run on a BD LSRII flow cytometer. The data was analyzed using DIVA software and the % of cells expressing IL-17 or IFNγ was obtained by gating on CD45^+^/βTcR^+^/CD4^+ ^T cells. B) RT-PCR was performed on RNA isolated from one lung lobe from individual WT or IL-6^-/- ^mice (n = 4/group) exposed to saline or *S. rectivirgula *using primers specific for IL-17A and IL-17F. The housekeeping gene β-actin was used as an internal control.

T cells are required for granuloma formation in HP and although there is an increase in Th17 cells in the T-bet^-/- ^mice which correlates with disease severity, it is unclear whether Th17 cells are required for granuloma formation. The differentiation of Th17 cells requires TGFβ and IL-6 during activation of naïve T cells and in the absence of IL-6 Th17 cells do not differentiate. We can detect increases in IL-6 in the BALF 18 hrs following exposure with *S. rectivirgula *(saline = 3 +/- 4pg/ml vs. *S. rectivirgula *= 1,180 +/- 163 pg/ml; *p *< 0.05). We also detected an increase in TGFβ in the BALF of mice exposed to *S. rectivirgula *(saline = 143 +/- 5 pg/ml vs. *S. rectivirgula *= 205 +/- 26 pg/ml; *p *< 0.05). To inhibit the development of Th17 cells during HP we used mice deficient in IL-6 and exposed them to *S. rectivirgula *for 3 weeks to measure the development of granulomas. IL-6^-/- ^mice developed a similar degree of alveolitis compared to WT mice with no significant differences in the cellular composition of the alveolitis (data not shown). There were changes in the cellular composition of the infiltrating immune cells (defined by expression of CD45) into the interstitial lung tissue (Table 3). IL-6^-/- ^mice exposed to *S. rectivirgula *demonstrated a decrease in the % of CD4+ T cells as compared to the WT mice (IL-6^-/- ^= 11+/-2.6% CD4^+ ^T cells vs WT = 18+/-4% CD4^+ ^T cells; *p *< 0.05) whereas, the CD8^+ ^T cell population did not differ between the two groups. There was also a significant increase in the % of monocytes/macrophages in the IL-6^-/- ^mice exposed to *S. rectivirgula *as compared to WT mice (IL-6^-/- ^= 38 +/- 3.9 mon/mac vs. WT = 19 +/- 2.6 mon/mac; *p *< 0.05). To determine whether Th1 cells migrated into the lungs and confirm that IL-6 was necessary for Th17 cell development in this model, intracellular cytokine staining was performed on lung cells stimulated with PMA and ionomycin (Figure 6A). The results demonstrate that there is a decrease in the % of Th17 cells as well as IFNγ^+^/IL-17^+ ^T cells in the lungs of IL-6^-/- ^mice following *S. rectivirgula *exposure compared to WT mice. However, there appeared to be a corresponding increase in the % of IFNγ^+^/IL-17^- ^T cells (Th1 cells) in the IL-6^-/- ^mice compared to WT mice. Although cells other than Th17 cells can also produce IL-17 in this model, the lack of Th17 cells resulted in a decrease in IL-17A and IL-17F mRNA in the lungs of IL-6^-/- ^mice exposed to *S. rectivirgula *(Figure 6B). Despite the decrease in Th17 cells (and IL-17 mRNA) in the lungs of IL-6^-/- ^mice, H&E staining of lung sections indicated that there was still granuloma formation in the lungs similar to WT mice, indicating that Th1 cells are sufficient for granuloma formation and Th17 cells are not absolutely required (Figure 4A & B). These results suggest that while IL-6 plays an important role in determining the balance of Th1 vs. Th17 cells in the disease, the development of a Th1 response is sufficient for granuloma formation.

**Table 3:** Cellular composition of infiltrating immune cells in WT and IL-6^-/- ^mice following *S. rectivirgula *exposure

	**CD4**^**+ **^**T cells**^**a **^**(% +/- SD)**	**CD8**^**+ **^**T cells**^**b **^**(% +/- SD)**	**Mon/Mac**^**c **^**(% +/- SD)**	**NK cells**^**d **^**(% +/- SD)**
**WT/*S. rectivirgula***	18 +/- 4.0	6 +/- 1.3	19 +/- 2.6	5 +/- 1
**IL-6**^**-/- **^**/*S. rectivirgula***	11 +/- 2.6*	6 +/- 1.8	38 +/- 3.9*	6 +/-0.9

C57BL/6 or IL-6^-/- ^mice (n = 5/group) were exposed to *S. rectivirgula *(150 μg) 3 times a week for three weeks and flow cytometry performed on lung cells isolated from individual mice to determine the cellular composition of the infiltrating cells using antibody to various cell surface markers and run on a BD LSRII flow cytometer. The data was analyzed using FACS DIVA software.

^a ^CD4^+ ^T cells were identified as βTcR^+^/CD45^+^/CD4^+^

^b ^CD8^+ ^T cells were identified as βTcR^+^/CD45^+^/CD8^+^

^c ^Moncytes/macrophages were identified as CD45^+^/CD11b^+^/Gr1^-^

^d ^NK cells were identified as CD45^+^/NK1.1^+^

*p < 0.05 compared to WT/*S. rectivirgula *group

## Discussion

Previous studies have demonstrated that IFNγ is necessary for the development of granulomas in HP and the results from these studies demonstrate that the transcription factor T-bet plays a critical role in controlling the severity of HP. Our in vitro studies demonstrated that IL-18 in combination with either IL-12 or IL-15 stimulated production of IFNγ from neutrophils, which we had previously identified as a cell source of IFNγ during the innate response to *S. rectivirgula *[15]. The synergistic actions of IL-18 with IL-12 or IL-15 may reflect the effect of IL-18 on cytokine receptor expression by neutrophils or the need for binding of multiple transcription factors to the IFNγ gene. One of the transcription factors activated by IL-18 is T-bet and our results show that IFNγ production induced by *S. rectivirgula *is completely dependent on T-bet. The results from these in vitro studies led us to propose that both IL-18 and T-bet would be required for IFNγ production in vivo during HP. Our results using IL-18^-/- ^mice revealed that in the absence of IL-18, IFNγ mRNA and protein were reduced compared to WT mice during the innate immune response to *S. rectivirgula*. These results suggest that IL-18 plays a role in the induction of IFNγ during the innate response to *S. rectivirgula*, although it is possible that other cytokines such as IL-12 also contribute to IFNγ production. IL-18 has been demonstrated to stimulate IFNγ production by stimulating expression of T-bet, however, we could not detect differences in the expression of T-bet mRNA in the IL-18^-/- ^mice compared to WT exposed mice (data not shown). A kinetic study of T-bet expression is needed to determine the optimal time point to examine the effect of IL-18 deficiency on its expression; the time points we examined may be too long after an exposure. Also, it is likely that other cytokines such as IL-12 also contribute to T-bet expression in this model and therefore the absence of IL-18 alone may not be sufficient to reveal a decrease in its expression. IL-18 is important for inducing a proinflammatory cascade and in addition to IFNγ, IL-17 mRNA was also decreased. Ruth *et al *[25] also found a decrease in IL-17 when IL-18^-/- ^mice (in comparison to WT mice) were injected with zymosan in a murine arthritis model and suggested that IL-18 may also play a role in regulating Th17 responses. In our model we cannot determine whether IL-18 directly induces IL-17 or does so through the induction of other cytokines. IL-17 is associated with strong neutrophil responses and can induce the neutrophil chemokine MIP-2. However, despite the decrease in IL-17mRNA the level of alveolitis (and neutrophil recruitment) was similar in the IL-18^-/- ^mice compared to WT mice exposed to *S. rectivirgula*. Our previous studies demonstrated that *S. rectivirgula *directly induces MIP-2 and we did not detect a decrease in MIP-2 mRNA in the IL-18^-/- ^mice compared to WT mice and therefore, at least during the innate immune response, IL-17 does not appear to be necessary for neutrophil recruitment or MIP-2 production. The effects of IL-18 signaling deficiency were restricted to the innate immune response to *S. rectivirgula*; longer exposures did not result in any effects on several parameters of disease severity. IL-18^-/- ^mice did not differ from WT mice exposed to *S. rectivirgula *in the level of alveolitis, granuloma formation, or cytokine production following 3 weeks of exposures. These results suggest that IL-18 plays a role in IFNγ production (as well as IL-17 production) during the innate response to *S. rectivirgula*, however IL-18 is dispensable for IFNγ production as well as granuloma formation during later phases of the disease.

The production of IFNγ following *S. rectivirgula *stimulation in vitro is dependent on T-bet and our in vivo results confirmed this. In the absence of T-bet, IFNγ mRNA expression in the lung and protein in the BALF was reduced compared to WT mice during the innate immune response. Surprisingly, IL-17 mRNA was also reduced despite some reports suggesting that in the absence of IFNγ or T-bet, IL-17 levels increase [23,24]. Despite the decrease in IL-17, the levels of MIP-2 mRNA in the T-bet^-/- ^mice were similar to WT and IL-18^-/- ^mice again suggesting that IL-17 isn't contributing to MIP-2 production early in the disease course. Surprisingly, the T-bet^-/- ^mice exhibited decreased alveolitis compared to the WT or IL-18^-/- ^mice during both the early and late stages of the disease. The reason for this is not clear however, T-bet controls the expression of genes other than IFNγ, including adhesion molecules and chemokine receptors that may be involved in neutrophil recruitment in this model [26-28].

To determine the effect of T-bet on development of granulomas and fibrosis, mice were exposed to *S. rectivirgula *for longer time periods. Our results demonstrated that T-bet was not required for CD4+ T cell migration into the lung following exposure; however, the expression of CXCR3 was significantly reduced on CD4+ T cells from T-bet^-/- ^mice compared to WT mice. T-bet binds to the CXCR3 promoter and directly upregulates CXCR3 expression and therefore it is not surprising to see a decrease in CXCR3^+ ^T cells in the lungs of Tbet^-/- ^mice [27,28]. These results suggest that CXCR3 expression is not required for the migration of pathogenic T cells into the lung; studies are underway to identify the chemokine(s)/chemokine receptors that are responsible for Th17 cell recruitment into the lung in this model. To determine the effects of T-bet on IFNγ production and the development of the Th17 response, we examined T-bet^-/- ^mice that had been exposed to *S. rectivirgula *for 3 or 6 weeks. The results demonstrated that T-bet^-/- ^mice did not develop Th1 cells, as expected, and the level of IFNγ mRNA was reduced, although not completely absent. Therefore, during the later phases of the disease, IFNγ production also occurs via a T-bet-dependent pathway although there may be a contribution by T-bet-independent pathways. T-bet^-/- ^mice had approximately double the % of Th17 cells in the lung compared to WT mice exposed to *S. rectivirgula*. T-bet has been reported to inhibit Th17 cell development via IFNγ production as well as through RORγt repression [23,24,29]; however, IFNγ^-/- ^mice develop less severe disease following *S. rectivirgula *exposure suggesting that the effects of T-bet cannot be attributed to IFNγ production [13]. We have not detected significant differences in RORγt expression at the time points measured (data not shown) and studies are currently underway to determine the mechanism by which T-bet is suppressing Th17 development. The shift to a stronger Th17 response in the lungs of exposed T-bet^-/- ^mice correlated with an increase in granuloma formation and total collagen content indicating a more severe onset of fibrosis at 6 weeks of exposure. These results are in agreement with studies by Simonian et al [16] and Joshi et al [30] which suggested that Th17 cells are associated with disease severity in HP. Exposure of IL-17RA^-/- ^mice to *S. rectivirgula *resulted in decreased inflammation and fibrosis compared to WT mice suggesting a pivotal role for Th17 cells in the disease process [16]. Although the role of IL-17 and its related cytokines in fibrosis remain to be clarified, the correlation between increased IL-17 and collagen content in this model suggests that IL-17 may be a key player in fibrosis in HP. Wilson *et al *[31] reported that IL-17A is necessary for development of fibrosis in the murine bleomycin model. IL-10 was an important regulator of IL-17 in this model, IL-10^-/- ^mice have elevated IL-17A and develop more severe fibrosis which was inhibited in IL-10^-/-^/IL-17^-/- ^double knockout mice. There was a strong correlation with IL-1β expression and fibrosis in both the IL-10^-/- ^and IL-10^-/-^/IL-17^-/- ^mice exposed to bleomcyin suggesting that IL-1β may play a role in IL-17A dependent fibrosis. Simonian et al [32] demonstrated, using a *B. subtilis *model of HP, that IL-22 produced by γδ T cells acts in a protective manner, inhibiting CD4+ T cell migration into the lung and collagen deposition. Further studies are needed to investigate whether T-bet expression regulates IL-22 or IL-1β production in this model.

Although the Th17 response was associated with more severe disease, our results indicate that a Th1 response is not protective and can also lead to granuloma formation in HP. Th17 cell differentiation requires IL-6 and TGFβ and our results indicate that both are present in the lungs of mice exposed to *S. rectivirgula*. Mice that are deficient in IL-6 production develop a similar level of alveolitis compared to WT mice however there were differences in the cellular composition of the immune cells recruited into the lung interstitium. Following exposure to *S. rectivirgula *IL-6^-/- ^mice exhibited a decrease in the percentage of CD4^+ ^T cells and an increase in the percentage of monocytes/macrophages compared to WT mice. It is difficult to determine if the changes in the cellular composition in the IL-6^-/- ^mice is due to the lack of IL-6 or the switch from a Th17 dominant response to a Th1 dominant response. The IL-6^-/- ^mice had a significant decrease in Th17 cells and an increase in Th1 cells in the lungs following *S. rectivirgula *exposure compared to the WT mice which had predominantly Th17 cells. The WT mice exposed to *S. rectivirgula *had a population of IFNγ^+^/IL-17^+ ^T cells following PMA and ionomycin stimulation which we have consistently observed at several time points (data not shown). However, this population does not appear in response to ex vivo *S. rectivirgula *stimulation and the role of these cells in HP is unknown. In other models IL-17^+^/IFNγ^+ ^T cells have been suggested to play a role in pathogenicity; using the EAE murine model of MS, Murphy, et al. [33] demonstrated that these cells infiltrate the brain prior to the development of symptoms. Additionally, MOG specific T cells that were IL-17^+^/IFNγ^+ ^were able to activate microglia cells in vitro resulting in production of TNFα, IL-1β, and IL-6 which contribute to CNS inflammation. The decrease in Th17 cells in IL-6^-/- ^mice is expected to result in a decrease in IL-17 production and that was reflected by a decrease in IL-17A and IL-17F mRNA. However, other cells besides αβT cells make IL-17 in this model [34] and therefore IL-17 production was not completely inhibited in the IL-6^-/- ^mice. The results demonstrate that IL-6 is necessary for the development of a Th17 response in HP and IL-6 is critical in determining the balance between a Th1 and Th17 response. Although it has been reported that IL-6^-/- ^mice have an increase in Treg activity [35] we did not see evidence of an increase in Tregs in our model. The *S. rectivirgula *exposed IL-6^-/- ^mice had similar levels of alveolitis and granuloma formation as well as an increase in IFNγ^+ ^T cells compared to the WT mice. The decrease in Th17 cells was accompanied by an increase in the percentage of Th1 cells in the lungs and H&E staining revealed granuloma formation in the lungs of the IL-6^-/- ^mice demonstrating that a Th1 response will also result in pathology and that Th17 cells are not absolutely required for granuloma formation. Numerous experimental models have demonstrated that Th1 and Th17 cells co-exist during disease and even co-localize to areas of pathology suggesting that the cells may interact or regulate each other in still unknown ways [23,36,37]. Although the prevailing thought has been that Th17 cells are mediating pathology in some of these diseases it is becoming apparent that in some cases both Th1 and Th17 cells may cause disease. For example, Luger *et al *[38] demonstrated that the murine model of autoimmune uveitis (EAU) can be mediated by either Th1 or Th17 cells. Additionally, EAE was induced in naïve mice by transfer of antigen specific T cells modulated in vitro by IL-12 or IL-23 [39]. The T cells cultured in the presence of IL-12 demonstrated a Th1 phenotype and upon transfer into naïve mice induced EAE with CNS infiltrates consisting predominantly of monocytes and lymphocytes with few neutrophils. Whereas IL-23 cultured T cells retained a Th17 phenotype upon transfer into naïve mice and induced a neutrophil rich CNS infiltrate. Intriguingly, the two forms of EAE responded differently to anti-GM-CSF therapy; EAE was suppressed in mice receiving IL-23- modulated T cells but not in mice receiving IL-12- modulated T cells [39]. Similarly, pathology in HP may develop in the presence of either a Th1 or Th17 response and the balance between the two types of cells may explain why some patients develop more severe fibrotic disease. Furthermore, as in the EAE model, there may be significant differences in the response to treatment between a Th1 dominant HP pathology and a Th17 dominant pathology.

The results from these studies demonstrate that T-bet plays a critical role during the development of HP. During the innate immune response to *S. rectivirgula*, T-bet regulates IFNγ and IL-17 production contributing to the severity of alveolitis. However, during the later phases of the disease T-bet plays a protective role by regulating development of the Th17 response, granuloma formation and fibrosis. The results suggest that the protective effect of T-bet is due to inhibition of the Th17 response and is not mediated by the development of Th1 cells; shifting the response to a predominant Th1 response also resulted in lung pathology. HP is a complex disease which may not be easily categorized as a Th1 - or Th17 - mediated disease. Identifying factors and subsequently the mechanisms by which they regulate these responses may lead to the identification of targets for therapeutic purposes.

## Declaration of competing interests

The authors declare that they have no competing interests.

## Authors' contributions

SN performed the neutrophil IFNγ production studies and initiated the IL-18^-/- ^studies. MD contributed to the Tbet^-/- ^and IL-6^-/- ^expts. HA performed the Tbet^-/- ^studies (including IFNγ production), and the real-time PCRs and ELISAs for all the studies. EF designed the studies and participated in the IL-6^-/- ^and IL-18^-/- ^studies. All authors read and approved the final manuscript.
